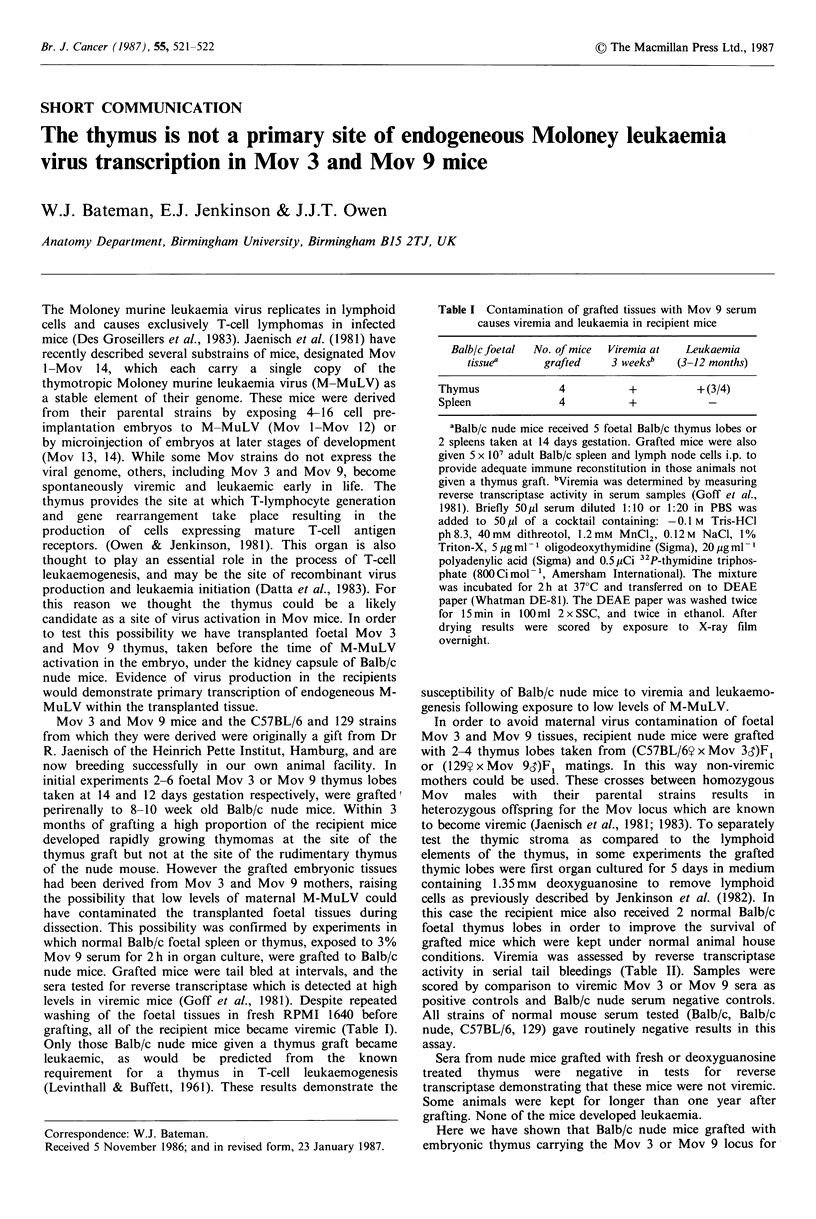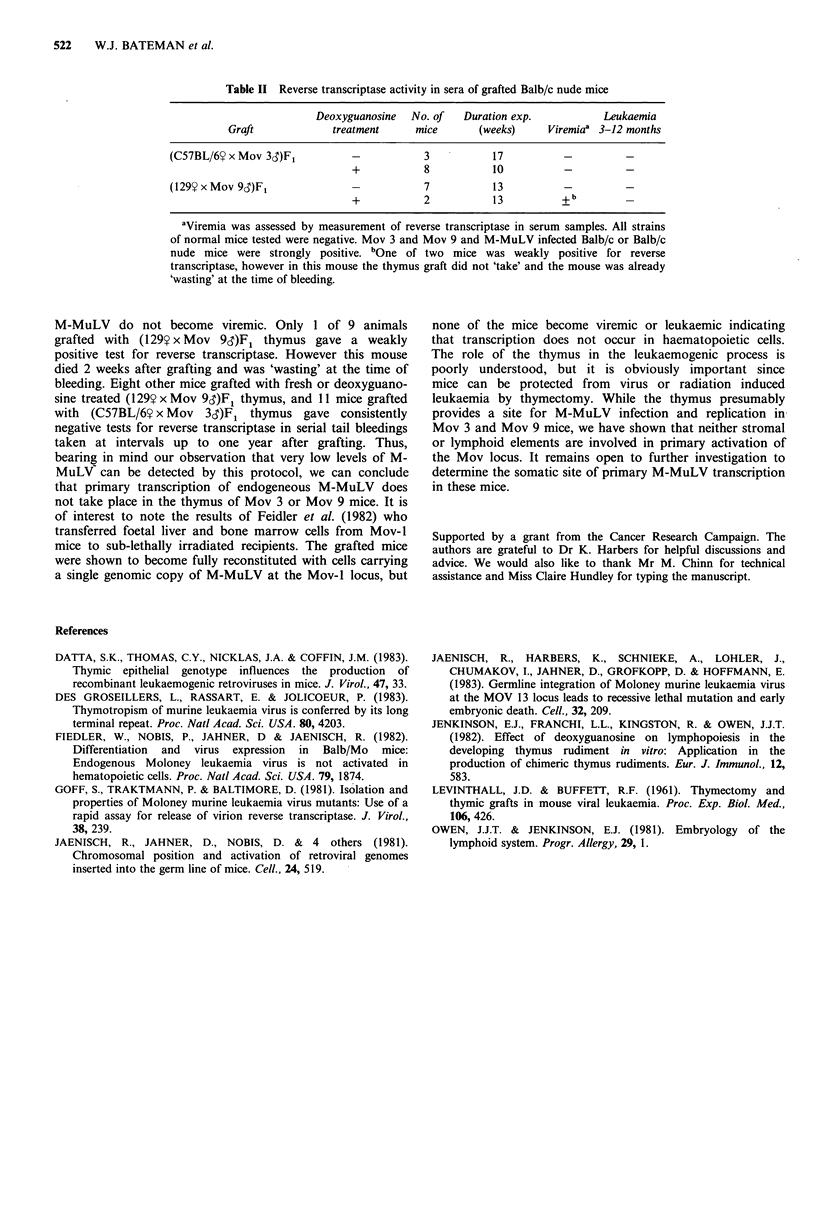# The thymus is not a primary site of endogenous Moloney leukaemia virus transcription in Mov 3 and Mov 9 mice.

**DOI:** 10.1038/bjc.1987.106

**Published:** 1987-05

**Authors:** W. J. Bateman, E. J. Jenkinson, J. J. Owen


*Br. J. Cancer (1987)*, **55**, 521–522 © The Macmillan Press Ltd., 1987

SHORT COMMUNICATION

### The thymus is not a primary site of endogeneous Moloney leukaemia virus transcription in Mov 3 and Mov 9 mice

W.J. Bateman, E.J. Jenkinson & J.J.T. Owen

*Anatomy Department, Birmingham University, Birmingham B15 2TJ, UK*

The Moloney murine leukaemia virus replicates in lymphoid cells and causes exclusively T-cell lymphomas in infected mice (Des Groseillers *et al.*, 1983). Jaenisch *et al.* (1981) have recently described several substrains of mice, designated Mov 1–Mov 14, which each carry a single copy of the thymotropic Moloney murine leukaemia virus (M–MuLV) as a stable element of their genome. These mice were derived from their parental strains by exposing 4–16 cell pre-implantation embryos to M–MuLV (Mov 1–Mov 12) or by microinjection of embryos at later stages of development (Mov 13, 14). While some Mov strains do not express the viral genome, others, including Mov 3 and Mov 9, become spontaneously viremic and leukaemic early in life. The thymus provides the site at which T-lymphocyte generation and gene rearrangement take place resulting in the production of cells expressing mature T-cell antigen receptors. (Owen & Jenkinson, 1981). This organ is also thought to play an essential role in the process of T-cell leukaemogenesis, and may be the site of recombinant virus production and leukaemia initiation (Datta *et al.*, 1983). For this reason we thought the thymus could be a likely candidate as a site of virus activation in Mov mice. In order to test this possibility we have transplanted foetal Mov 3 and Mov 9 thymus, taken before the time of M-MuLV activation in the embryo, under the kidney capsule of Balb/c nude mice. Evidence of virus production in the recipients would demonstrate primary transcription of endogeneous M-MuLV within the transplanted tissue.

Mov 3 and Mov 9 mice and the C57BL/6 and 129 strains from which they were derived were originally a gift from Dr R. Jaenisch of the Heinrich Pette Institut, Hamburg, and are now breeding successfully in our own animal facility. In initial experiments 2–6 foetal Mov 3 or Mov 9 thymus lobes taken at 14 and 12 days gestation respectively, were grafted perirenally to 8–10 week old Balb/c nude mice. Within 3 months of grafting a high proportion of the recipient mice developed rapidly growing thymomas at the site of the thymus graft but not at the site of the rudimentary thymus of the nude mouse. However the grafted embryonic tissues had been derived from Mov 3 and Mov 9 mothers, raising the possibility that low levels of maternal M-MuLV could have contaminated the transplanted foetal tissues during dissection. This possibility was confirmed by experiments in which normal Balb/c foetal spleen or thymus, exposed to 3% Mov 9 serum for 2 h in organ culture, were grafted to Balb/c nude mice. Grafted mice were tail bled at intervals, and the sera tested for reverse transcriptase which is detected at high levels in viremic mice (Goff *et al.*, 1981). Despite repeated washing of the foetal tissues in fresh RPMI 1640 before grafting, all of the recipient mice became viremic (Table I). Only those Balb/c nude mice given a thymus graft became leukaemic, as would be predicted from the known requirement for a thymus in T-cell leukaemogenesis (Levinthall & Buffett, 1961). These results demonstrate the

**Table I** Contamination of grafted tissues with Mov 9 serum causes viremia and leukaemia in recipient mice

| *Balb/c foetal tissue*[a] | *No. of mice grafted* | *Viremia at 3 weeks*[b] | *Leukaemia (3–12 months)* |
|---|---|---|---|
| Thymus | 4 | + | +(3/4) |
| Spleen | 4 | + | – |

[a]Balb/c nude mice received 5 foetal Balb/c thymus lobes or 2 spleens taken at 14 days gestation. Grafted mice were also given $5\times10^7$ adult Balb/c spleen and lymph node cells i.p. to provide adequate immune reconstitution in those animals not given a thymus graft. [b]Viremia was determined by measuring reverse transcriptase activity in serum samples (Goff *et al.*, 1981). Briefly 50 $\mu$l serum diluted 1:10 or 1:20 in PBS was added to 50 $\mu$l of a cocktail containing: –0.1 M Tris-HCl ph 8.3, 40 mM dithreotol, 1.2 mM $MnCl_2$, 0.12 M NaCl, 1% Triton-X, 5 $\mu g\,ml^{-1}$ oligodeoxythymidine (Sigma), 20 $\mu g\,ml^{-1}$ polyadenylic acid (Sigma) and 0.5 $\mu$Ci $^{32}P$-thymidine triphosphate (800 Ci $mol^{-1}$, Amersham International). The mixture was incubated for 2 h at 37°C and transferred on to DEAE paper (Whatman DE-81). The DEAE paper was washed twice for 15 min in 100 ml 2 × SSC, and twice in ethanol. After drying results were scored by exposure to X-ray film overnight.

susceptibility of Balb/c nude mice to viremia and leukaemogenesis following exposure to low levels of M-MuLV.

In order to avoid maternal virus contamination of foetal Mov 3 and Mov 9 tissues, recipient nude mice were grafted with 2–4 thymus lobes taken from (C57BL/6♀ × Mov 3♂)$F_1$ or (129♀ × Mov 9♂)$F_1$ matings. In this way non-viremic mothers could be used. These crosses between homozygous Mov males with their parental strains results in heterozygous offspring for the Mov locus which are known to become viremic (Jaenisch *et al.*, 1981; 1983). To separately test the thymic stroma as compared to the lymphoid elements of the thymus, in some experiments the grafted thymic lobes were first organ cultured for 5 days in medium containing 1.35 mM deoxyguanosine to remove lymphoid cells as previously described by Jenkinson *et al.* (1982). In this case the recipient mice also received 2 normal Balb/c foetal thymus lobes in order to improve the survival of grafted mice which were kept under normal animal house conditions. Viremia was assessed by reverse transcriptase activity in serial tail bleedings (Table II). Samples were scored by comparison to viremic Mov 3 or Mov 9 sera as positive controls and Balb/c nude serum negative controls. All strains of normal mouse serum tested (Balb/c, Balb/c nude, C57BL/6, 129) gave routinely negative results in this assay.

Sera from nude mice grafted with fresh or deoxyguanosine treated thymus were negative in tests for reverse transcriptase demonstrating that these mice were not viremic. Some animals were kept for longer than one year after grafting. None of the mice developed leukaemia.

Here we have shown that Balb/c nude mice grafted with embryonic thymus carrying the Mov 3 or Mov 9 locus for

Correspondence: W.J. Bateman.
Received 5 November 1986; and in revised form, 23 January 1987.

**Table II** Reverse transcriptase activity in sera of grafted Balb/c nude mice

| *Graft* | *Deoxyguanosine treatment* | *No. of mice* | *Duration exp. (weeks)* | *Viremia*[a] | *Leukaemia 3–12 months* |
|---|---|---|---|---|---|
| (C57BL/6♀ × Mov 3♂)$F_1$ | – | 3 | 17 | – | – |
| | + | 8 | 10 | – | – |
| (129♀ × Mov 9♂)$F_1$ | – | 7 | 13 | – | – |
| | + | 2 | 13 | ±[b] | – |

[a]Viremia was assessed by measurement of reverse transcriptase in serum samples. All strains of normal mice tested were negative. Mov 3 and Mov 9 and M-MuLV infected Balb/c or Balb/c nude mice were strongly positive. [b]One of two mice was weakly positive for reverse transcriptase, however in this mouse the thymus graft did not 'take' and the mouse was already 'wasting' at the time of bleeding.

M-MuLV do not become viremic. Only 1 of 9 animals grafted with (129♀ × Mov 9♂)$F_1$ thymus gave a weakly positive test for reverse transcriptase. However this mouse died 2 weeks after grafting and was 'wasting' at the time of bleeding. Eight other mice grafted with fresh or deoxyguanosine treated (129♀ × Mov 9♂)$F_1$ thymus, and 11 mice grafted with (C57BL/6♀ × Mov 3♂)$F_1$ thymus gave consistently negative tests for reverse transcriptase in serial tail bleedings taken at intervals up to one year after grafting. Thus, bearing in mind our observation that very low levels of M-MuLV can be detected by this protocol, we can conclude that primary transcription of endogeneous M-MuLV does not take place in the thymus of Mov 3 or Mov 9 mice. It is of interest to note the results of Feidler *et al.* (1982) who transferred foetal liver and bone marrow cells from Mov-1 mice to sub-lethally irradiated recipients. The grafted mice were shown to become fully reconstituted with cells carrying a single genomic copy of M-MuLV at the Mov-1 locus, but none of the mice become viremic or leukaemic indicating that transcription does not occur in haematopoietic cells. The role of the thymus in the leukaemogenic process is poorly understood, but it is obviously important since mice can be protected from virus or radiation induced leukaemia by thymectomy. While the thymus presumably provides a site for M-MuLV infection and replication in Mov 3 and Mov 9 mice, we have shown that neither stromal or lymphoid elements are involved in primary activation of the Mov locus. It remains open to further investigation to determine the somatic site of primary M-MuLV transcription in these mice.

Supported by a grant from the Cancer Research Campaign. The authors are grateful to Dr K. Harbers for helpful discussions and advice. We would also like to thank Mr M. Chinn for technical assistance and Miss Claire Hundley for typing the manuscript.

### References

DATTA, S.K., THOMAS, C.Y., NICKLAS, J.A. & COFFIN, J.M. (1983). Thymic epithelial genotype influences the production of recombinant leukaemogenic retroviruses in mice. *J. Virol.*, **47,** 33.

DES GROSEILLERS, L., RASSART, E. & JOLICOEUR, P. (1983). Thymotropism of murine leukaemia virus is conferred by its long terminal repeat. *Proc. Natl Acad. Sci. USA.* **80,** 4203.

FIEDLER, W., NOBIS, P., JAHNER, D & JAENISCH, R. (1982). Differentiation and virus expression in Balb/Mo mice: Endogenous Moloney leukaemia virus is not activated in hematopoietic cells. *Proc. Natl Acad. Sci. USA.* **79,** 1874.

GOFF, S., TRAKTMANN, P. & BALTIMORE, D. (1981). Isolation and properties of Moloney murine leukaemia virus mutants: Use of a rapid assay for release of virion reverse transcriptase. *J. Virol.*, **38,** 239.

JAENISCH, R., JAHNER, D., NOBIS, D. & 4 others (1981). Chromosomal position and activation of retroviral genomes inserted into the germ line of mice. *Cell.*, **24,** 519.

JAENISCH, R., HARBERS, K., SCHNIEKE, A., LOHLER, J., CHUMAKOV, I., JAHNER, D., GROFKOPP, D. & HOFFMANN, E. (1983). Germline integration of Moloney murine leukaemia virus at the MOV 13 locus leads to recessive lethal mutation and early embryonic death. *Cell.*, **32,** 209.

JENKINSON, E.J., FRANCHI, L.L., KINGSTON, R. & OWEN, J.J.T. (1982). Effect of deoxyguanosine on lymphopoiesis in the developing thymus rudiment *in vitro*: Application in the production of chimeric thymus rudiments. *Eur. J. Immunol.*, **12,** 583.

LEVINTHALL, J.D. & BUFFETT, R.F. (1961). Thymectomy and thymic grafts in mouse viral leukaemia. *Proc. Exp. Biol. Med.*, **106,** 426.

OWEN, J.J.T. & JENKINSON, E.J. (1981). Embryology of the lymphoid system. *Progr. Allergy*, **29,** 1.